# In vitro hepatotoxicity of *Petasites hybridus* extract (Ze 339) depends on the concentration, the cytochrome activity of the cell system, and the species used

**DOI:** 10.1002/ptr.6516

**Published:** 2019-10-20

**Authors:** Kristina Forsch, Verena Schöning, Greta Marie Assmann, Christin Moser, Beate Siewert, Veronika Butterweck, Jürgen Drewe

**Affiliations:** ^1^ Preclinical Research Max Zeller Söhne AG Romanshorn Switzerland; ^2^ Department of Biology University of Konstanz Konstanz Germany

**Keywords:** cytotoxicity, HepaRG, HepG2, H‐4‐II‐E, *Petasites hybridus*, species differences

## Abstract

Ze 339, a CO_2_ extract prepared from the leaves of *Petasites hybridus,* possesses antispasmodic and anti‐inflammatory effects and is proven to be effective in the treatment of allergic rhinitis. To study possible hepatotoxic effects of Ze 339, its main constituents and metabolites, a series of in vitro investigations were performed. Furthermore, different reconstituted fractions of extract (petasins and fatty acid fraction) were examined in three in vitro test systems using hepatocytes: Two human cell lines, with lower and higher activity of cytochrome P450 enzymes (HepG2, HepaRG) as well as a rodent cell line with high cytochrome P450 activity (H‐4‐II‐E), were used. Metabolic activity, assessed by the WST‐1 assay, was chosen as indicator of cytotoxicity. To assess potential bioactivation of Ze 339 compounds, metabolic experiments using S9 fractions from rats, dogs, and humans and isolated cytochromes (human/rat) were performed, and the formation of reactive metabolites was assessed by measuring cellular concentrations of glutathione and glutathione disulphide.

Our data revealed that the cytotoxicity of Ze 339, its single constituents, and main metabolites depends on the concentration, the cytochrome activity of the cell system, and the species used.

## Graphical abstract

The hepatoxicity of the Petasites hybridus leaf extract Ze _339_ was investigated in different in vitro test systems using human (HepG_2_ or HepaRG cells) or rat (H‐_4_‐II‐E cells) hepatocytes. Cytotoxicity of Ze _339_, its single constituents and main metabolites depends on the concentration, the cytochrome activity of the cell system and the species used. Cytotoxicity was in vitro only observed after concentrations, which are 1000‐fold higher than those obtained after administration of clinically recommended doses of Ze _339_ in patients.

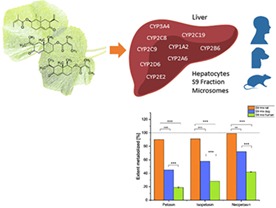

## 1 BACKGROUND

*Petasites hybridus* (L.) gaertn., b.mey. & scherb. (common name: butterbur) is an herbaceous perennial plant belonging to the Asteraceae family. Herbal extracts of *P. hybridus* are traditionally used in European phytotherapy for the treatment of various ailments including migraines and tension headaches, spasms of the urogenital and digestive tracts, asthma, allergic rhinitis, allergic skin disease, gastric ulcers, and skin wounds (Anonymus, 2001). Nowadays, *P. hybridus* has been proven to be effective in the treatment of migraines and allergic rhinitis (Dumitru et al., 2011; Grossman & Schmidramsl, 2001; Schapowal & Petasites study group, 2002, 2004; Schapowal & Study Group, 2005).

**Figure 1:**
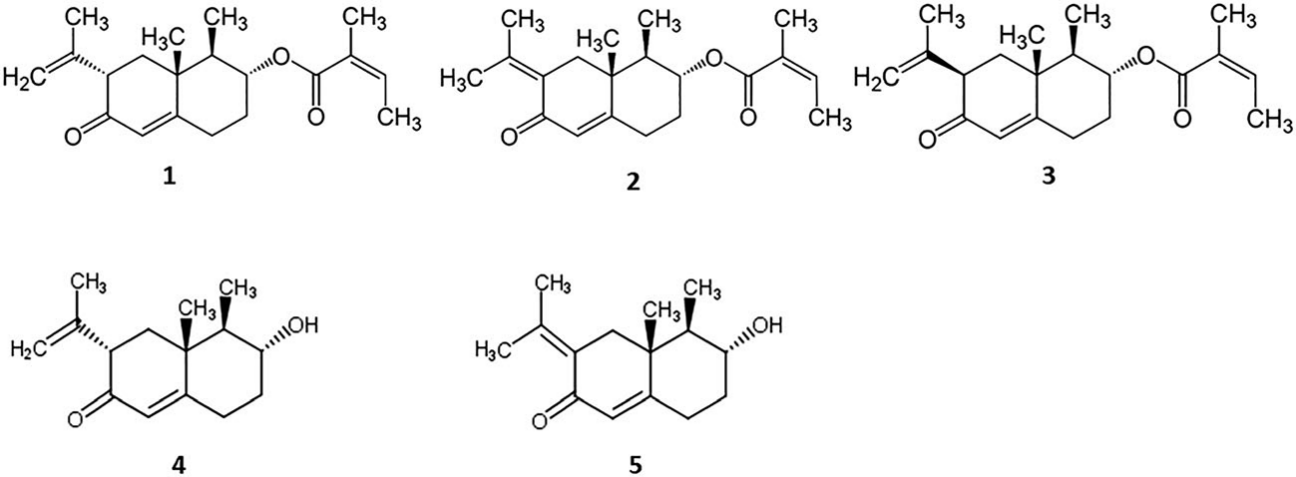
Chemical structures of petasin (**1**), isopetasin (**2**), neopetasin (**3**), and the metabolites petasol (**4**) and isopetasol (**5**)

*P. hybridus* extract Ze 339 is a CO_2_ extract prepared from the leaves of the plant. The main active constituents of butterbur are the petasin isomers petasin, isopetasin, and neopetasin (Figure 1), which have been shown to inhibit the biosynthesis of leukotrienes associated with spasmolytic activity (O. A. Thomet, Schapowal, Heinisch, Wiesmann, & Simon, 2002; O. A. R. Thomet, Wiesmann, Schapowal, Bizer, & Simon, 2001).

Nine cases of severe clinical hepatotoxicity of a medicinal product containing *P. hybridus* root extract (Petadolor/Dolomed) for migraine prophylaxis occurred between November 2001 and February 2002 using therapeutic doses (Evers, 2009). This led to the subsequent withdrawal of the Marketing Authorization of medicinal products containing the root extract in Switzerland in 2004 (Swissmedic, 2004).

However, up to now, no evidence is available regarding the hepatotoxicity of the leaf extract Ze 339. Thus, it was the aim of this study to determine possible in vitro hepatotoxic effects of Ze 339, the petasin isomers petasin, isopetasin, and neopetasin as well as the metabolites petasol and isopetasol.

The analysis focused on three different in vitro test systems of hepatocytes: two human hepatic cell lines, with lower and higher activity of cytochrome P450 enzymes (HepG2 and HepaRG, respectively, (Andersson, Kanebratt, & Kenna, 2012; Guillouzo et al., 2007)) and a rat cell line (H‐4‐II‐E [Fujimura, Murakami, Miwa, Aruga, & Toriumi, 2012; Westerink, Stevenson, & Schoonen, 2008]) were used. As a marker for cytotoxicity, the WST‐1 assay was chosen.

To assess potential bioactivation, metabolic experiments using S9 fractions from Sprague Dawley rats, beagle dogs and humans, and isolated cytochromes (human and rat) were performed. The formation of reactive metabolites was indirectly assessed by measuring the cellular concentrations of glutathione and glutathione disulphide levels after treatment with Ze 339.

## 2 METHODS

### 2.1 Chemicals

Minimum essential medium (MEM)‐Glutamax media, Dulbecco's modified Eagle medium‐Glutamax media, William's medium, sodium pyruvate, MEM nonessential amino acids, penicillin/streptomycin, l‐glutamine, 2‐(4‐(2‐hydroxyethyl)‐1‐piperazinyl)‐ethansulphoic acid. Glutamax‐I (100X), Type I rat tail collagen, and fetal bovine serum were obtained from Gibco (Carlsbad, Californian, USA).

Positive controls (digitonin), cytochromes inhibitors (ketoconazole, omeprazole, sulfaphenazole, montelukast, α‐naphthoflavone, quinidine, ticlopidine, clomethiazole, and xanthotoxin), fatty acids (palmitic acid, stearic acid, oleic acid, linoleic acid, γ‐linolenic acid, linolenic acid, *cis*‐4,7,10,13,16,19‐docosahexaenoic acid, and arachidonic acid), ammonium acetate, acetonitrile, dimethyl sulfoxide (DMSO), and ethanol were purchased from Sigma Aldrich (Switzerland). All chemicals were purchased with the highest grade available. Enzymes S9 fraction from human, dog (beagle), and rat (Sprague Dawley) were supplied from Invitrogen (UK). NADPH solution A (20‐fold) and solution B (100‐fold) were obtained from BD Biosciences (Switzerland) and Promega (Switzerland). Ammonia solution was supplied from Merck (Germany). Isolated cytochrome P450 enzymes and phosphate buffer were obtained from BD Gentest (Switzerland). Petasin (purity 92.82%), isopetasin (purity 96.18%), and neopetasin (purity 94.77%) were obtained from HWI Analytik GmbH (Germany). Petasol (purity>98%) was purchased from Adipogen (Switzerland), and isopetasol (purity >95%) was a kind gift from Dr. E. Küsters (Basel, Switzerland). *P. hybridus* native extract Ze 339 was provided by Max Zeller Söhne AG (Switzerland).

WST‐1 kit was purchased from BioVision (Switzerland). Sulforhodamine B kit was obtained from Sigma Aldrich (Switzerland). GSH‐Glo™ Glutathione kit was purchased from Promega Corporation (Switzerland).

### 2.2 Cell cultures

Human hepatocellular carcinoma HepG2 and rodent (*Rattus norvegicus*) H‐4‐II‐E cell lines were purchased from ATCC (UK). The human hepatocellular carcinoma HepaRG‐cell line was obtained from Invitrogen (Switzerland).

H‐4‐II‐E and HepG2 cells were maintained, differentiated, and treated in 75‐cm^2^ culture flask (Semadeni, Switzerland) as adherent cells in MEM‐Glutamax media with 10% (v/v) fetal bovine serum, 0.5‐mM sodium pyruvate, 1× MEM nonessential amino acids, and 1% (v/v) penicillin/streptomycin. Frozen, undifferentiated HepaRG‐cells were thawed and seeded according to manufacturer's manual to differentiate to mature cells.

### 2.3 *P. hybridus* extract, single constituents, and mixtures

Ze 339, a commercial subcritical (Herrero, Cifuentes, & Ibanez, 2006) CO_2_ extract from *P. hybridus* leaves (drug‐extraction ratio 50–100:1), was manufactured by Max Zeller Söhne AG, Switzerland. It is marketed as Tesalin® film‐coated tablets for the treatment of allergic rhinitis in Switzerland and other countries since 2003 (Dumitru et al., 2011; Grossman & Schmidramsl, 2001; Schapowal & Group, 2002; Schapowal & Petasites Study group, 2004; Schapowal & Study Group, 2005). One tablet is standardized to contain 8 mg total petasins. The batch of Ze 339 tested comprised of two main fractions: petasin fraction (in the investigated batch: petasin 18.9%, isopetasin 15.4%, and neopetasin 2.1%) and fatty acid fraction (34.0%). The remaining 29.6% contains other constituents (e.g., essential oils, sterols, minerals, and vitamins).

The extract Ze 339, its major active constituents petasin, isopetasin, and neopetasin, and petasol and isopetasol were tested to evaluate their contribution to possible cytotoxic effects. The latter compounds were precursors of petasin isomers, to a minor extent constituents of Ze 339 as well as metabolites. However, a rigorous species dependent formation was not studied in vivo in different species.

To investigate whether the petasin and the fatty acid fractions contribute to the cytotoxic effects, the following mixtures were prepared having the same concentration of single constituents as the extract, Ze 339: (a) *Petasin* *mixture*: containing petasin, isopetasin, and neopetasin; (b) fatty acid mixture: containing fatty acid components (e.g., palmitic acid, stearic acid, oleic acid, linoleic acid, γ‐linolenic acid, linolenic acid, cis‐4,7,10,13,16,19‐docosahexaenoic acid, and arachidonic acid); (c) Reconstituted extract: composed of the petasin mixture and the fatty acid mixture. Ze 339, all compounds, and mixtures were dissolved in DMSO (stock solution) and further diluted cell culture medium. The final assay concentration of DMSO did not exceed 0.5%.

### 2.4 Concentrations applied

Ze 339 (containing 36.4% of total petasins) the mixtures and the reconstituted extract were applied in final concentrations between 0.02 and 100 μg/ml (containing up to 113 μM total petasins). For toxicity evaluation of the single constituents, a concentration range between 3.9 and 250 μM was used.

### 2.5 Cytotoxicity

HepG2 and H‐4‐II‐E cells were differentiated for 24 hr. HepaRG cells were directly seeded in precoated 96‐well microtiter plates and were incubated in William's medium with general purpose supplements according to manufacturer's manual until adherence. All cells were treated with Ze 339 (up to 100 μg/ml) or petasin, isopetasin, neopetasin, petasol, isopetasol, petasin mixture, fatty acid mixture, and reconstituted extracts (up to 250 μM) for 4 hr.

In the three cell lines, cytotoxicity was defined as a decrease in metabolic activity of ≥20% in the WST‐1 assay. At the end of the experiment, 10 μl WST‐1 reagent was added to each well. Plates were then incubated for 2 hr to allow for the reduction of WST‐1 reagent. Absorbance was measured using a microplate absorbance reader (Infinite M 200, Tecan Trading Ltd., Switzerland) at 450 nm, reference wavelength of 620 nm. Due to some matrix effect, the lowest concentration (0.02 μg/ml for Ze 339 and 3.9 μM for single constituents and mixtures) of each substance tested was defined as 100% metabolic activity. Results were expressed as percentage of the control metabolic activity, and data were presented as *mean values* ± *SEM* (*n* = 3–6).

### 2.6 Metabolism of isolated constituents

The metabolism of petasin, isopetasin, and neopetasin was investigated using three different S9 fractions originating from three different species (isolated from rat, dog, and human liver homogenates containing cytochrome P450 isoforms) and isolated cytochromes of two different species (human and rat; rat analogues of the respective human cytochromes were used). For the expression of the different cytochromes, a baculovirus expression system was used according to supplier's manual. The S9 fractions or respectively single P450 cytochromes were used to mimic the liver metabolism by in vitro incubation.

Positive controls were specific for each of the investigated cytochrome and species. For human cells: phenacetin (CYP1A2), efavirenz (CYP2B6), amodiaquine (CYP2C8), diclofenac (CYP2C9), dextromethorphan (CYP2D6), testosterone (CYP3A4), and for rat cells phenacetin (CYP1A2), testosterone (CYP2A1, CYP3A1, CYP3A2), diclofenac (CYP2C6) testosterone (CYP2C11), and P‐nitrophenol (CYP2E1) were used.

The assay was carried out in triplicate in 1.5 ml glass vials with a final concentration of 20 μM of petasin, isopetasin, and neopetasin. The incubation was performed in a 37°C warm water bath. S9 fractions were thawed slowly on ice. Four different approaches were pursued by testing: (a) petasin, neopetasin, and isopetasin before (0 min) and after 60 min incubations with S9 fraction; (b) petasin, neopetasin, and isopetasin without S9 fraction after 60 min incubation; (c) vehicle as negative controls; and (d) cofactors of S9 fraction after 60‐min incubation.

A mixture of NADPH regeneration systems A (20‐fold) and B (100‐fold), phosphate buffer, and H_2_O was added, preincubated at 37°C, and shaken gently for 5 min. Afterwards, the recommended amount between 10 and 40 pmol/ml of enzyme or enzyme mixture was added according to manufacturer's instruction and incubated for further 60 min at 37°C and gently shaken. Afterwards, the reactions were stopped by addition of acetonitrile (400 μl). For the control sample, acetonitrile was added at 0 min incubation. The samples were centrifuged for 3 min at 10,000 × g. The supernatant was transferred to high‐performance liquid chromatography vials and analyzed by ultra‐performance liquid chromatography mass spectrometry (UPLC/MS). The UPLC analysis was performed with an UPLC Acquity H Class from Waters (Waters Corporation, Switzerland) including a quaternary high‐pressure gradient pump, an automatic sample injector, and a column thermostat. Chromatographic separation was achieved on an Acquity BEH C18, 1.7 μl, 50 × 2.1 mm column (Waters, Switzerland). The mobile phase consisted of 0.1% formic acid (A) and acetonitrile (B). The initial gradient condition was 10% (B) followed by a step to 90% (B) until 6.0 min. The column temperature was adjusted to 40°C. The flow rate was 0.5 ml/min and the injection volume 5 μl. The mass spectrometry was performed in positive electrospray mode using an ACQUITY QDa mass detector (Waters Corporation, Switzerland). The single ion recording was set to 317.2 Da.

### 2.7 Inhibition of single cytochromes

To investigate whether metabolic activation by human phase I enzymes—at least in part—contributes to the cytotoxic effect, Ze 339 (concentrations up to 100 μg/ml) was coincubated in HepaRG cells for 4 hr with different single cytochromes inhibitors: ketoconazole (CYP3A4), sulfaphenazole (CYP2C9), montelukast (CYP2C8), omeprazole (CYP2C19), α‐naphthoflavone (CYP1A2), quinidine (CYP2D6), xanthotoxin (CYP2A6), chlormethiazole (CYP2E1), and ticlopidine (CYP2B6)—(each at a concentration of 10 μM). Inhibition of metabolic activity was evaluated with the WST‐1 assay using Ze 339 without inhibitor as a control.

### 2.8 Effects on GSH and GSSG

The kinetics of glutathione (GSH) depletion and glutathione disulfide (GSSG) formation after incubation with Ze 339 was determined in HepaRG cells. Cells were seeded with a density of 20 × 10^4^ cells in 100 μl medium per well in a 96‐well assay plate. Afterwards, Ze 339 (concentrations up to 100 μg/ml) and the solvent (0.5% DMSO) were added. Then, cells were incubated for further 4 hr at 37°C with 5% CO_2_ atmosphere. The amounts of GSH and GSSG were determined using GSH/GSSG‐Glo™ assay, a luminescence‐based assay for detection and quantification of total glutathione (GSH + GSSG), GSSG and GSH/GSSG ratios in cultured cells. The assay was performed according to manufacturer's instruction (Tecan Infinite M200, Switzerland).

### 2.9 Statistics

Data were analyzed by descriptive analysis and graphically displayed by Origin 2017 Software (Originlab Corp., USA). Concentration‐response analysis was done using the sigmoidal Hill equation: E(*t*) = E_0 −_ E_max_ × C(*t*)^N^/(IC_50_^N^ + C(*t*)^N^), where E is the effect at time *t*, E_0_ the effect at baseline (time = 0), IC_50_ the concentration yielding 50% of the maximal effect (E_ma_x), and *N* denotes the sigmoidicity coefficient (Origin 2017 Software). For statistical analysis, analysis of variance with Dunnett's multicomparison test was used to correct for multiplicity of testing (IBM SPSS Statistics software, version 25, IBM Corp., USA). The level of significance was ^*^ *p* < .05, ^**^ *p* < .01 and ^***^ *p* < .001; NS = *no significance*.

Chromatographic data analysis was performed with Empower 3 software (Waters Corp., Switzerland).

## 3 RESULTS

### 3.1 Cytotoxicity of Ze 339, petasin, isopetasin, and neopetasin

**Figure 2:**
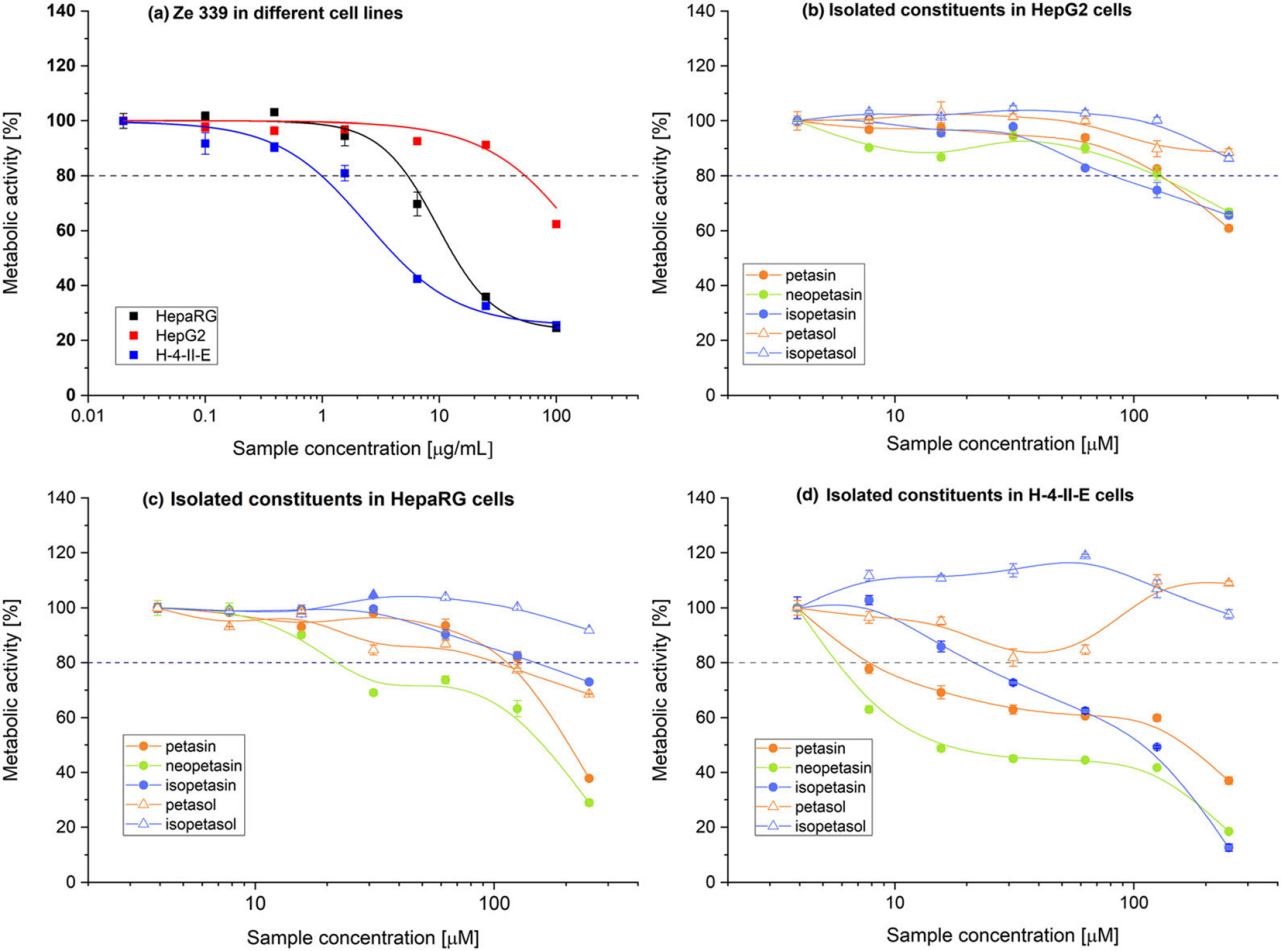
Mean metabolic activity (WST‐1 test) of Ze 339 and isolated constituents (petasin, neopetasin, isopetasin, petasol, and isopetasol) after administration in different cell lines. (a) Effects of Ze 339 in HepaRG, HepG2, and H‐4‐II‐E cells (*n* = 6); (b) effects of isolated constituents in HepG2 cells (*n* = 3); (c) effects of isolated constituents in HepaRG cells (*n* = 3); and (d) effects of isolated constituents in H‐4‐II‐E cells (*n* = 3) [Colour figure can be viewed at http://wileyonlinelibrary.com]

The hepatotoxic activity of Ze 339 was examined in hepatic cell lines from human (HepG2 and HepaRG) and rat (H‐4‐II‐E) donors (Figure 2a).

There was a species‐dependent decrease in cellular metabolic activity in hepatocytes: Most sensitive were rat H‐4‐II‐E cells, followed by human HepaRG cells, and then human HepG2 cells (EC_50_ = 2.4, 9.62, and > 100 μg/ml, respectively, Figure 2a–d).

To identify the constituents responsible for the cytotoxic effect of Ze 339, the experiments were repeated using only single constituents of Ze 339 (either petasin, isopetasin, or neopetasin) or their metabolites (petasol and isopetasol). The concentrations of the isolated constituents tested in the individual assays highly exceeded the concentration of these substances in the extract Ze 339.

In HepG2 cells, a moderate inhibition of the metabolic activity was achieved with petasin, isopetasin, and neopetasin at the highest concentration tested (250 μM). At this concentration, metabolic activity decreased down to 60.9%, 65.6%, and 66.8%, respectively, when compared with the reference concentration (3.9 μM; Figure 2b). The main metabolites petasol and isopetasol did not show cytotoxic effects.

In HepaRG, petasin and neopetasin exhibited a strong cytotoxic effect with a decrease of metabolic activity to 37.7% and 28.9%, respectively, at 250 μM. Isopetasin and petasol were moderately cytotoxic (metabolic activity 73% and 68.6%, respectively). Isopetasol was not cytotoxic at the highest concentration (Figure 2c).

In H‐4‐II‐E cells, neopetasin (EC_50_ = 15.75 μM) was more toxic compared with petasin (EC_50_ = 132 μM) and isopetasin (EC_50_ = 110 μM). The concentration‐response curves of petasin and neopetasin were comparable with a rapid decrease in metabolic activity at low concentrations, a plateau at medium concentrations with only slight changes, and again a rapid decrease at the highest concentration. In comparison, the concentration‐response curve of isopetasin displays a rather constant decrease in metabolic activity with increasing concentrations. At medium concentrations (15.6–62.5 μM), petasol induced a slight decrease in metabolic activity, but at concentrations ≥125 μM, the metabolic activity increased up to 109%. In contrast, isopetasol showed an increase in metabolic activity from 7.8 to 125 μM; however, at the highest concentration, virtually no change was observed (Figure 2d).

### 3.2 Cytotoxicity of the petasin mixture, fatty acids mixture, and reconstituted extract

**Figure 3:**
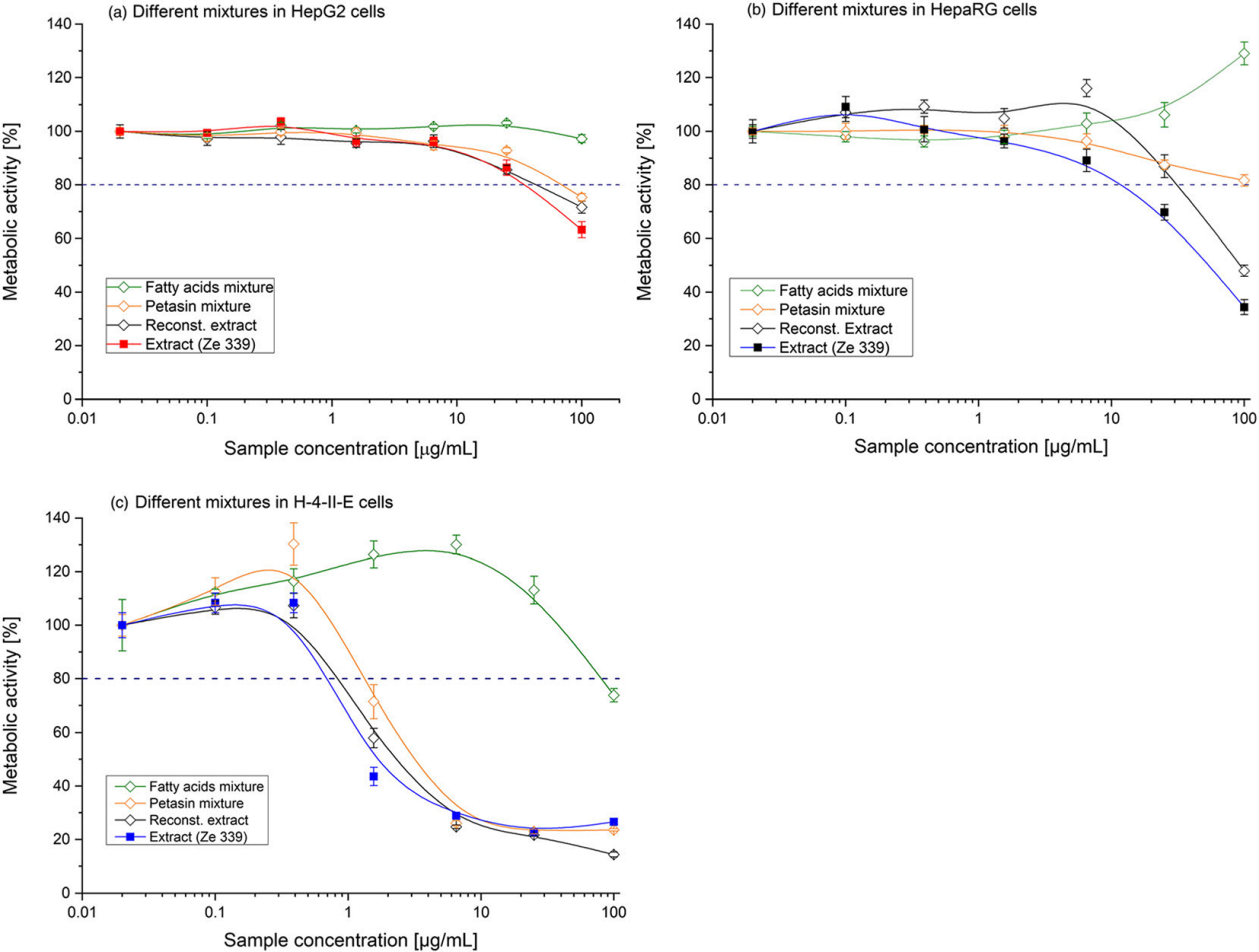
Mean effects of Ze 339, petasin mixture, fatty acid mixture, and the reconstituted extract (a) in HepG2 cells, (b) in HepaRG cells, and (c) in H‐4‐II‐E cells (*n* = 3 − 6) [Colour figure can be viewed at http://wileyonlinelibrary.com]

In HepG2 cells, the fatty acids mixture did not decrease metabolic activity. Petasin mixture, reconstituted extract, and Ze 339 showed comparable, mild cytotoxicity, in the highest concentration a decrease in metabolic activity down to 60–80% of the control (Figure 3a).

In HepaRG cells, neither the fatty acid mixture alone nor the petasin mixture inhibited metabolic activity. The combination of these mixtures, the reconstituted extract, however, showed a moderate reduction in metabolic activity (30–50%) similar to Ze 339 in HepaRG cells (Figure 3b).

In H‐4‐II‐E cells, the toxic effects of all treatments seemed to be intensified: The petasin mixture, the reconstituted extract as well as Ze 339 showed marked reduction in metabolic activity (below 30%). Even the fatty acid mixture indicated a mild 26% reduction in metabolic activity (Figure 3c).

### 3.3 Metabolism of Ze 339 and single constituents

**Figure 4:**
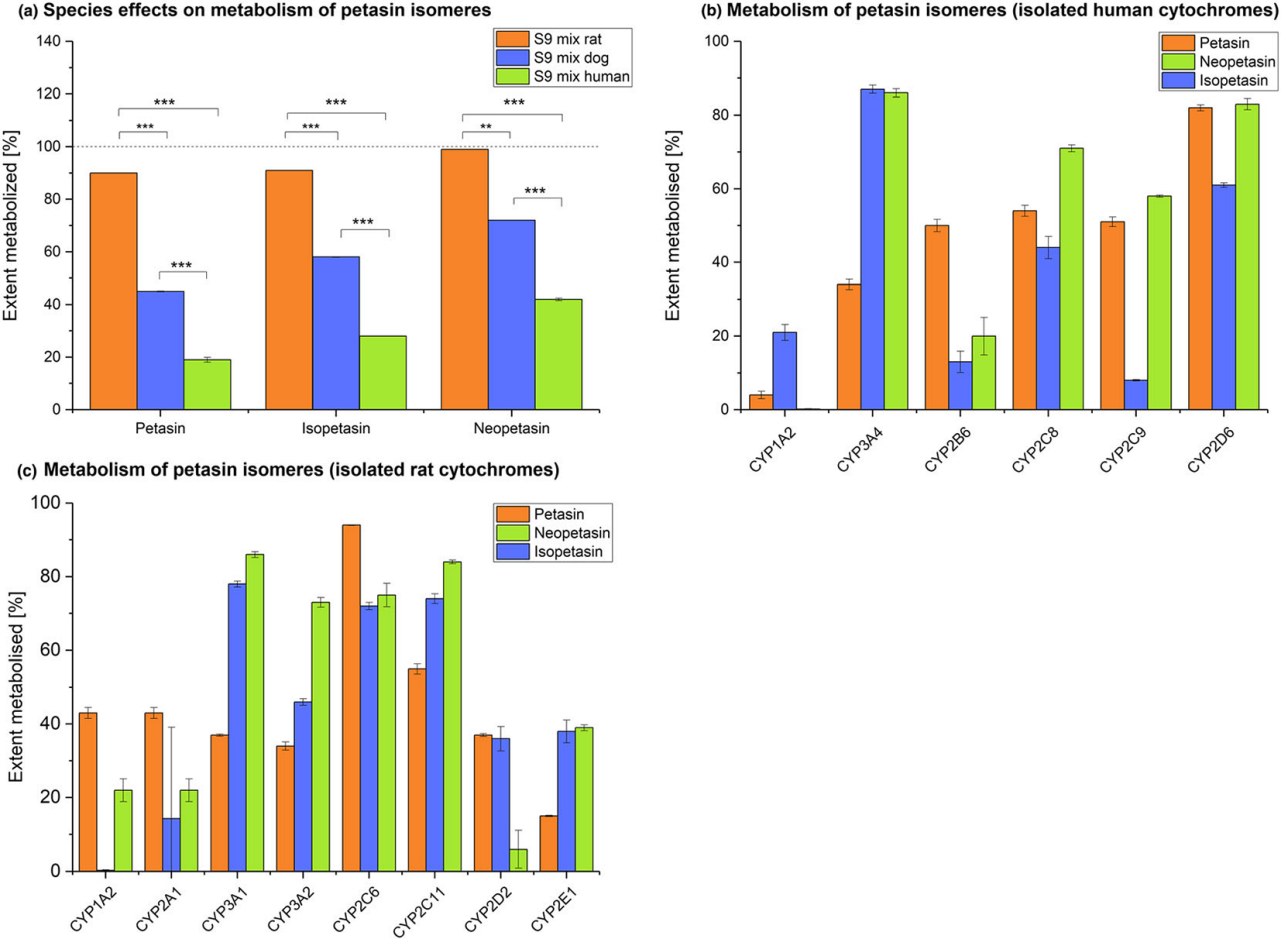
(a) *Mean* ± *SEM* extent of metabolism (percent dose) of petasin, isopetasin, and neopetasin after application of 20 μM and incubation over 60 min. Incubation of petasin isomers with S9 fractions from different species (rats, dogs, and human) using water as negative control (*n* = 3). For each of the petasin isomers, there was a significant difference in the extent of metabolism between the S9 fractions from species of donors (^***^ *p* < .001, ^**^ *p* < .01, analysis of variance with Dunnett's multicomparison test). (b,c) *M* ± *SEM* metabolism of petasin, isopetasin, and neopetasin by cytochromes from different species after application of 20 μM and incubation over 60 min and after application of 20 μM and incubation over 60 min. Incubation (*n* = 3) with (b) different single human CYP P450 isoforms and (c) different single rat CYP P450 isoforms (*n* = 3) [Colour figure can be viewed at http://wileyonlinelibrary.com]

After incubation of S9 fractions from different species with the three isolated constituents petasin, isopetasin, and neopetasin, a significant (*p* < .01 or *p* < .001) species‐dependent metabolism after 60 min incubation was observed with the following rank order: rat>dog>human (Figure 4a). Incubation with either solvent or only cofactors of S9 fraction as negative controls did not show relevant metabolism of petasin, isopetasin, and neopetasin (with solvent: <1%, <1%, and <1% and with cofactor: 10%, <1%, and <1%, respectively, data not shown).

The incubation with single human cytochromes revealed that petasin was mainly metabolized by CYP2D6 (80.2%) and to an intermediate extent by CYP3A4 (34%), CYP2B6 (50%), CYP2C8 (54%), and CYP2C9 (51%). Virtually no metabolism was observed for CYP1A2 (4%). However, for isopetasin and neopetasin, the cytochromes mainly involved in their metabolism were CYP3A4 (87% and 86%), CYP2D6 (61% and 83%), and CYP2C8 (44% and 71%). For CYP2C9, diverging effects were observed (8% and 58%), and for CYP2B6, virtually no metabolism (13% and 20%) was noted (Figure 4b).

Incubation with rat cytochromes revealed a different pattern of metabolism compared with human cytochromes: For CYP1A2, petasin was higher (43% vs. 4%), and neopetasin (22% vs. <1%) and isopetasin (<1% vs. 21%) lower metabolized, respectively. CYP3A showed a similar pattern: petasin/neopetasin/isopetasin metabolism with CYP3A4 (human) was 34%/87%/86% versus CYP3A1 (rat): 37%/78%/86% versus CYP3A2 (rat): 34%/46%/73%. CYP2C‐mediated metabolism was in general higher with rat than with human cytochromes. Extend of metabolism was 54%/44%/71% for CYP2C8 (human) and 51%/8%/58% for CYP2C9 (human) versus 94%/72%/75% for CYP2C6 (rat) and 55%/74%/84% for CYP2C11 (rat). Most pronounced was the difference for the CYP2D family: Extend of petasin/neopetasin/isopetasin metabolism was 82%/61%/83% for CYP2D6 (human) versus 37%/36%/7% for CYP2D2 (rats). CYP2B6 (human) and CYP2E1 (rat) could not be compared between species because the respective analogue of the other species was not available to us. (Figure 4c).

**Figure 5:**
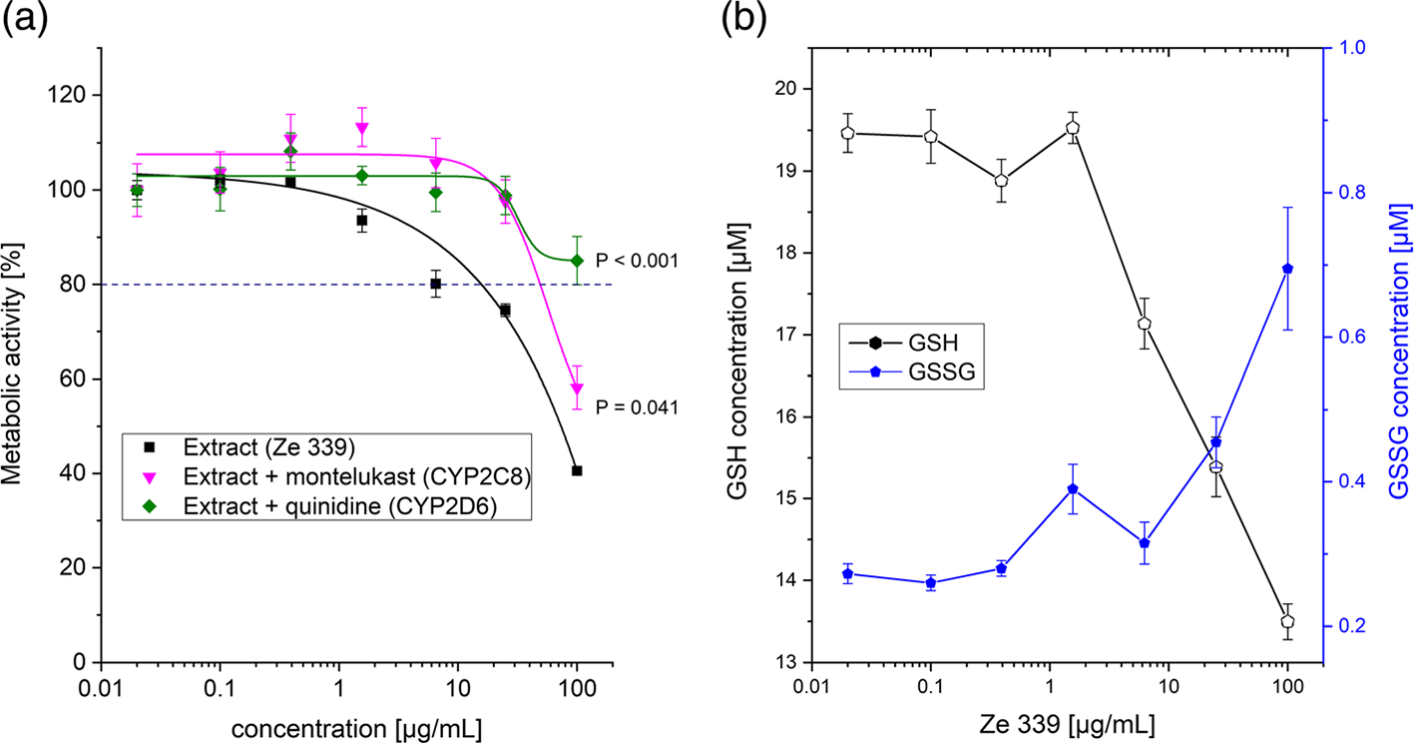
(a) Mean metabolic activity (±SEM) after administration of Ze 339 and cytochrome inhibitors in HepaRG cells. The cells were incubated for 4 hr at 37°C. Inhibition of CYP2D6 and CYP2C8 significantly attenuated Ze 339 toxicity. Statistical analysis of the highest dose by analysis of variance and Dunnett's multicomparison test. In this figure, only data were shown, where a significant effect of CYP inhibition on Ze 339 toxicity was observed (*n* = 6 − 7). (b) Effects of Ze 339 on the intracellular concentrations of GSH and GSSG formation in HepaRG cells. The cells were incubated for 4 hr at 37°C. Data are expressed as *Mean* ± *SEM*), (*n* = 3) [Colour figure can be viewed at http://wileyonlinelibrary.com]

Positive controls for each of the isolated cytochromes showed an extent of metabolism between 31.2% (CYP1A2) and 100% (CYP2C9) for human cytochromes and between 23.1% (CYP2D2) and 100% (CYP2B1) for rat cytochromes (data not shown). In HepaRG cells, the effect of cytochrome inhibition on the toxic effects of Ze 339 was investigated (Figure 5a); addition of quinidine (CYP2D6 inhibitor) and montelukast (CYP2C8 inhibitor) attenuated (*p* < .001 and *p* = .041, respectively); most of the cytotoxic effect of Ze 339 demonstrated for the highest concentration studied (100 μg/ml). No significant effect on Ze 339 toxicity was observed for the other CYP inhibitors (see Figure S1).

The results of the latter experiment indicate that several cytochromes contribute at least partly to the toxic effect, presumably by formation of reactive metabolites (biotransformation). The biggest contribution was shown for CYP2D6 (Figure 5a).

### 3.4 Determination of glutathione depletion and glutathione disulphide formation

The investigations of the effect of Ze 339 on the cellular redox status revealed a concentration‐dependent decrease in GSH concentration from 19.46 μM at 0.02 μg/ml down to 13.54 μM at 100 μg/ml Ze 339 (Figure 5b). Correspondingly, the GSSG formation was concentration‐dependently increased after treatment with Ze 339.

## 4 DISCUSSION

The cytotoxicity of Ze 339 and its single constituents petasin, isopetasin, neopetasin, isopetasol, and petasol was shown to be dependent on the concentration used, the cytochrome activity, and species. Comparison of two human cell lines (HepG2 and HepaRG) with different activities of cytochrome P450 enzymes revealed that Ze 339 and its single constituents petasin, isopetasin, neopetasin, isopetasol, and petasol exerted higher toxicity in cells having a higher cytochrome activity. Furthermore, Ze 399 and petasin, isopetasin, and neopetasin were more toxic in rat cells (H‐4‐II‐E), which are known to have a high expression of cytochrome P450 (Fujimura et al., 2012; Westerink et al., 2008) than in both human cell lines. Experiments analyzing the metabolism of petasin, isopetasin, and neopetasin after incubation with different S9 fractions from Sprague Dawley rats and beagle dogs and humans showed that the extent of metabolism is species‐specific for all three petasins with the following clear rank order: rat>dog>human.

This rank order complies with animal studies with Ze 339, which established a no adverse effect level of 60 mg/kg/day in 13‐ and 26‐week studies in rats, whereas in a 4‐week study in dogs, a no adverse effect level of 750 mg/kg/day was observed (data on file). The data suggest a species effect in the sense that rats are relatively more sensitive to the toxic effects of Ze 339 than dogs and humans.

The observed results of the in vitro and the in vivo experiments suggest the involvement of a concentration‐ and, respectively, dose‐dependent metabolic degradation and bioactivation of Ze 339 and/or its constituents; however, other not yet identified factors may also contribute. Considering the involvement of cytochromes, the metabolic conversion into a toxin (“toxification”) is dependent not only on the activity of these enzymes but also on the types of constitutively expressed cytochromes. Besides daily dose (≥100 mg) and lipophilicity (LogP≥3), bioactivation was identified as a significant risk factor for the development of drug‐induced hepatotoxicity (Chen, Borlak, & Tong, 2013; Yu et al., 2014). The importance of these risk factors was also recently confirmed by us in larger dataset (Hammann, Schöning, & Drewe, 2019). This may explain the observed species‐specific effects in preclinical in vivo studies, with rats being the most sensitive species, followed by dogs, and the good tolerability profile in clinical studies and post‐marketing observations.

Experiments with isolated cytochromes showed that the metabolic pathway of petasin, isopetasin, and neopetasin involves different cytochromes, further explaining the species‐specific sensitivity to the toxic effects of Ze 339. Inhibition of different cytochromes in HepaRG showed an attenuation of the toxic effects, which was pronounced for CYP2D6 and to a minor extend CYP2C8. One limitation of the study is the use of HepaRG cells, which is an immortalized hepatic cell line that retains many characteristics of primary human hepatocytes (Antherieu et al., 2010). Exposure of HepaRG cells to potential inducers resulted in the induction of most of the major drug metabolizing CYP isoenzymes (Aninat et al., 2006; Kanebratt & Andersson, 2008). Therefore, the European Medicines Agency (EMA, 2012) classified HepaRG cells as supportive model to cultured (fresh or cryopreserved) human hepatocytes. However, HepaRG cells are generated from a donor carrying a polymorphism for CYP2D6 (Guillouzo et al., 2007), which leads to a lower expression of this enzyme in HepaRG cells compared with, for example, primary hepatocytes (Kanebratt & Andersson, 2008; Zanelli, Caradonna, Hallifax, Turlizzi, & Houston, 2012), and therefore, CYP2D6 activity might be underestimated. On the other hand, because CYP2D6 inhibition by quinidine resulted in attenuated toxicity of Ze 339 in HepaRG cells, this still emphasizes the involvement of this cytochrome enzyme in the development of the hepatotoxicity.

The hypothesis of metabolic bioactivation of Ze 339 is further supported by the observation that Ze 339 administration concentration‐dependently decreases cellular GSH concentration and GSSG formation, which may indicate the response to oxidative stress.

Comparison of the toxicity of different fraction mixtures and combinations thereof showed that the petasin mixture (36.4% of the extract) alone was less toxic than Ze 339 itself. The fatty acid mixture alone (~30% of the extract) did not exert any toxic effects. However, the combination of the petasin and the fatty acid mixture (reconstituted extract) had comparable toxic effects as Ze 339 itself. One possible explanation for this synergistic effect is that the fatty acid mixture may increase the solubility of the highly lipophilic petasin isoforms (XlogP of about 4.5, https://pubchem.ncbi.nlm.nih.gov/) in the hydrophilic extracellular medium, thus, enhancing the cellular uptake of the fatty acid/petasin complex.

The clinically recommended daily dose of Ze 339 for the treatment of allergic rhinitis is two to three tablets containing up to 80–120 mg of the extract (corresponding to 16–24 mg petasins). In a clinical study in 21 healthy volunteers (Vogt & Will‐Shahab, 1999), two tablets were administered resulting in mean petasin plasma concentrations of 25.5 ng/ml. We could demonstrate in our present experiments that administration of the petasin mixture at a concentration above 25 μg/ml resulted in only a subtoxic reduction of metabolic activity in HepaRG cells by 12.5 % (Figure 3b). Thus, using clinically recommended doses of Ze 339 in patients, the resulting peak plasma concentration is 1,000‐fold lower, indicating that toxic effects of Ze 339 occur only after high, supra‐pharmacologic doses.

Since the Marketing Authorization of a medicinal product containing *Petasites* leaf extract Ze 339 (Tesalin®) in Switzerland in March 2003, no severe hepatotoxic reactions were observed in six controlled clinical trials and five post‐marketing surveillance studies. Ze 339 is currently (2019) marketed in Switzerland, Korea, and Mexico. Furthermore, a patient exposure of approximately 38 million defined daily doses was estimated. On the basis of the recommended clinical regimen of two tablets daily, an exposure of approximately 104,000 patient years with Ze 339 can be assumed. Overall, no index case of hepatotoxicity was detected.

Therefore, the *P. hybridus* leaf extract Ze 339 can be regarded as safe if used in the clinically recommended dose regime of two to three tablets per day. This was recently acknowledged by the Swiss health authority, as Tesalin® was switched from prescription to the nonprescription status in 2018.

## CONFLICT OF INTEREST

K. F., V. S., C. M., B. S., V. B., and J. D. are employees of Max Zeller Söhne AG. GA declares no conflict of interest.

## Supporting information

Data S1. Supporting information.
