# Supplementary material for: In vitro hepatotoxicity of Petasites hybridus extract (Ze 339) depends on the concentration, the cytochrome activity of the cell system, and the species used
Source: Phytother Res. 2019 Oct 20;34(1):184–92. doi: 10.1002/ptr.6516 (PMC7004140; doi:10.1002/ptr.6516)
Supplement: Supplementary file 1 — Data S1. Supporting information. [file PTR-34-184-s001.docx]

***In vitro* hepatotoxicity of *Petasites hybridus* extract (Ze 339) depends on the concentration, cytochrome activity of the cell system and the species used**

Kristina Forsch^1^, Verena Schöning^1^, Greta Marie Assmann^2^, Christin Moser^1^, Beate Siewert^1^, Veronika Butterweck^1^, Jürgen Drewe^1^

^1^Max Zeller Söhne AG, CH-8590 Romanshorn, Switzerland

^2^University of Konstanz, D-78464 Konstanz, Germany

**Short title:** Mechanisms of hepatotoxicity of Ze 339

***) Address of Correspondence**

Prof. Dr. Juergen Drewe

Preclinical Research

Max Zeller Söhne AG

Seeblickstrasse 4

CH-8590 Romanshorn

Switzerland

Phone: +41 79 695 6895

Email: [juergen.drewe@zellerag.ch](mailto:juergen.drewe@zellerag.ch)

Supplemental information





**Fig. S1.** Mean metabolic activity (± SEM) after administration of Ze 339 and cytochrome inhibitors in HepaRG cells**.** The cells were incubated for 4 h at 37 °C. Statistical analysis of the highest dose by analysis of variance und Dunnett’s multicomparison test. In this figure, only data were shown, where a significant effect of CYP inhibition on Ze 339 toxicity was observed (n=6-7).
